# Supplementary material for: Factors associated with phylogenetic clustering of hepatitis C virus, mainly among people who inject drugs who access HIV prevention services in South Africa, 2016–2017
Source: PLoS One. 2025 Dec 1;20(12):e0336614. doi: 10.1371/journal.pone.0336614 (PMC12668479; doi:10.1371/journal.pone.0336614)
Supplement: S1 Table — (DOCX) [file pone.0336614.s001.docx]

**S1 Table**. Substance use and sex practice of participants with Core-E2 sequences

| Characteristic  Total n (%) | Overall  (n = 141) | Durban  (n = 35) | Pretoria  (n = 59) | Cape Town  (n = 47) |
| --- | --- | --- | --- | --- |
| Substance use |  |  |  |  |
| Heroin | 97 (68.8) | 30 (85.7) | 32 (54.2) | 35 (74.5) |
| Methamphetamine | 40 (28.4) | 2 (5.7) | 2 (3.4) | 36 (76.6) |
| Shared needle at last injection | 19 (13.5) | 4 (11.4) | 7 (11.9) | 8 (17.0) |
| New needle at last injection | 82 (58.2) | 26 (74.3) | 32 (54.2) | 24 (51.1) |
| Injecting ≥ 4 times per day | 78 (55.3) | 21 (60.0) | 31 (52.5) | 26 (55.3) |
| Injected for a year | 114 (80.9) | 29 (82.9) | 47 (79.7) | 38 (80.9) |
| Sex practice |  |  |  |  |
| Condom at last penial-vaginal sex | 18 (12.8) | 4 (11.4) | 8 (13.6) | 6 (12.8) |
| Sexually active in the last month | 41 (29.1) | 8 (22.9) | 12 (20.3) | 21 (44.7) |
|  |  |  |  |  |
|  |  |  |  |  |
