# Supplementary material for: Factors associated with phylogenetic clustering of hepatitis C virus, mainly among people who inject drugs who access HIV prevention services in South Africa, 2016–2017
Source: PLoS One. 2025 Dec 1;20(12):e0336614. doi: 10.1371/journal.pone.0336614 (PMC12668479; doi:10.1371/journal.pone.0336614)
Supplement: S2 Table — Participants with HCV genotype 1a or 3a were included in the analysis. (DOCX) [file pone.0336614.s002.docx]

**S2 Table.** Characteristics of participants with Core-E2 sequence in a cluster, not in a cluster, and overall. Participants with HCV genotype 1a or 3a were included in the analysis.

| **Characteristics** | **Total**  **(n = 141)** | **Not in a cluster**  **(n = 63)** | **In a cluster**  **(n = 78)** | ***P-value*** |
| --- | --- | --- | --- | --- |
| **Age (years)** |  |  |  | **0.045 *** |
| ≥ 29 | 91 (64.5) | 35 (55.6) | 56 (71.8) |  |
| < 29 | 50 (35.5) | 28 (44.4) | 22 (28.2) |  |
| **HCV viral load**  **(log IU)** |  |  |  | 0.969 |
| ≥ 6 log IU | 96 (68.1) | 43 (68.1) | 53 (68.0) |  |
| < 6 log IU | 45 (31.9) | 20 (31.8) | 25 (32.1) |  |
| **Gender** |  |  |  | 0.563 |
| Male | 130 (92.2) | 59 (93.7) | 71 (91.0) |  |
| Female | 11 (7.8) | 4 (6.4) | 7 (9.0) |  |
| **HIV infection** |  |  |  | 0.511 |
| Positive | 43 (30.5) | 21 (33.3) | 22 (28.2) |  |
| Negative | 98 (69.5) | 42 (66.7) | 56 (71.8) |  |
| **HBV infection** |  |  |  | 0.512 |
| Positive | 10 (7.1) | 3 (4.8) | 7 (9.0) |  |
| Negative | 131 (93.0) | 60 (95.2) | 71 (91.0) |  |
| **Race** |  |  |  | **0.000 *** |
| White | 59 (41.8) | 24 (38.1) | 35 (44.9) |  |
| Black | 46 (32.6) | 30 (47.6) | 16 (20.5) |  |
| Mixed ancestry | 32 (22.7) | 5 (7.9) | 27 (56.9) |  |
| **Missing | 4 (2.8) | 4 (6.3) | 0 (0) |  |
| **HCV genotype** |  |  |  | 0.754 |
| 1a | 89 (63.1) | 40 (63.5) | 49 (62.8) |  |
| 3a | 49 (34.8) | 20 (31.8) | 29 (37.2) |  |
| **Missing | 3 (2.13) | 3 (4.8) | 0 (0) |  |
| **City** |  |  |  | **0.000 *** |
| Pretoria | 59 (41.8) | 32 (50.8) | 27 (34.6) |  |
| Cape Town | 47 (33.3) | 9 (14.3) | 38 (48.7) |  |
| Durban | 35 (24.8) | 22 (34.9) | 13 (16.7) |  |
| **Housing** |  |  |  | 0.511 |
| Homeless | 86 (61.0) | 39 (61.9) | 47 (60.3) |  |
| Shelter/flat | 36 (25.5) | 14 (22.2) | 22 (28.2) |  |
| * Missing | 19 (13.5) | 10 (15.9) | 9 (11.5) |  |
| **Injected for a year** |  |  |  | 0.276 |
| **Yes** | 114 (80.9) | 51 (81.0) | 63 (80.8) |  |
| No | 8 (7.0) | 2 (3.2) | 6 (7.7) |  |
| Missing | 19 (13.5) | 10 (15.9) | 9 (11.5) |  |
| **Injecting ≥ 4 times per day** |  |  |  | 0.736 |
| Yes | 78 (55.3) | 33 (52.4) | 45 (57.7) |  |
| No | 44 (31.2) | 20 (31.7) | 24 (30.8) |  |
| * Missing | 19 (13.5) | 10 (15.9) | 9 (11.5) |  |
| **New needle at last injection** |  |  |  | **0.189** |
| Yes | 82 (58.2) | 39 (61.9) | 43 (55.1) |  |
| No | 40 (28.4) | 14 (22.2) | 26 (33.3) |  |
| * Missing | 19 (13.5) | 10 (15.9) | 9 (11.5) |  |
| **Shared needle at last injection** |  |  |  | **0.130** |
| Yes | 19 (13.5) | 5 (7.9) | 14 (17.9) |  |
| No | 99 (70.2) | 14 (22.2) | 52 (66.7) |  |
| * Missing | 23 (16.3) | 11 (17.5) | 12 (15.4) |  |
|  |  |  |  |  |
| **Condom at last penial-vaginal sex** |  |  |  | 1.000 |
| Yes | 18 (12.8) | 8 (12.7) | 10 (12.8) |  |
| No | 104 (73.8) | 45 (71.4) | 59 (75.6) |  |
| * Missing | 19 (13.5) | 10 (15.9) | 9 (11.5) |  |
| **Sexually active in the last month** |  |  |  | 0.754 |
| Yes | 41 (29.1) | 17 (27.0) | 24 (30.8) |  |
| No | 81 (57.4) | 36 (57.1) | 45 (57.7) |  |
| * Missing | 19 (13.5) | 10 (15.9) | 9 (11.5) |  |
|  |  |  |  |  |

* Missing indicates variables with missing information

** Missing indicates variables with omitted information due to small numbers
