# Supplementary material for: Factors associated with phylogenetic clustering of hepatitis C virus, mainly among people who inject drugs who access HIV prevention services in South Africa, 2016–2017
Source: PLoS One. 2025 Dec 1;20(12):e0336614. doi: 10.1371/journal.pone.0336614 (PMC12668479; doi:10.1371/journal.pone.0336614)
Supplement: S3 Table — (DOCX) [file pone.0336614.s003.docx]

**S3 Table.** Univariate and multivariate logistic regression analysis of factors associated with being in a phylogenetic cluster for participants with HCV genotype 1a or 3a.

| **Characteristics** | **Overall**  **(n = 141)** | **Not in a cluster**  **(n = 63)** | **In a cluster**  **(n = 78)** | **Odds ratio** | **95% CI** | ***p*** | **Adjusted odds ratio** | **95% CI** | ***p*** |
| --- | --- | --- | --- | --- | --- | --- | --- | --- | --- |
|  |  |  |  |  |  |  |  |  |  |
| Shared needle | 19 (13.5) | 5 (7.9) | 14 (17.9) | 2.53 | 0.85-7.56 | 0.096 | 3.43 | 0.97-12.15 | **0.056** |
| New needle | 82 (58.2) | 39 (61.9) | 43 (55.1) | 0.59 | 0.27-1.30 | 0.191 |  |  |  |
| Age ≥ 31 | 76 (53.9) | 29 (46.0) | 47 (60.1) | 1.78 | 0.91-3.48 | 0.093 | 2.19 | 0.96-5.00 | 0.063 |
| **Race** |  |  |  |  |  |  |  |  |  |
| White |  |  |  | 1.00 |  |  | 1.00 |  |  |
| Black | 46 (32.6) | 30 (47.6) | 16 (20.5) | 0.37 | 0.16-0.81 | **0.014** |  |  |  |
| Mixed ancestry | 32 (22.7) | 5 (7.9) | 27 (56.9) | 3.70 | 1.25 -10.97 | **0.018** | 5.77 | 1.80-18.48 | **0.003** |
| **City** |  |  |  |  |  |  |  |  |  |
| Durban |  |  |  | 1.00 |  |  | 1.00 |  |  |
| Pretoria | 59 (41.8) | 32 (50.8) | 27 (34.6) | 1.43 | 0.61-3.36 | 0.415 |  |  |  |
| Cape town | 47 (33.3) | 9 (14.3) | 38 (48.7) | 7.14 | 2.63-19.40 | **0.000** |  |  |  |

In the multivariate analysis, one factor was positively associated with HCV phylogenetic clustering – mixed-ancestry race (aOR 5.77, 95% CI 1.80 – 18.48, *p 0.003)*. Mixed ancestry race was associated with clustering when adjusted for age ≥31 and sharing a needle at the last injection. The odds of being in a cluster were 6x if the participant was of mixed ancestry. This result was similar to the analysis conducted with age ≥29. However, the *p* values of sharing a needle at the last injection and age ≥31 were borderline, not significant.
