# Supplementary material for: Factors associated with phylogenetic clustering of hepatitis C virus, mainly among people who inject drugs who access HIV prevention services in South Africa, 2016–2017
Source: PLoS One. 2025 Dec 1;20(12):e0336614. doi: 10.1371/journal.pone.0336614 (PMC12668479; doi:10.1371/journal.pone.0336614)
Supplement: S4 Table — Analysis exclusively for PWID. (DOCX) [file pone.0336614.s004.docx]

**S4 Table.** Univariate and multivariate logistic regression analysis of factors associated with being in a phylogenetic cluster for participants with HCV genotype 1a or 3a. Analysis exclusively for PWID.

| **Characteristics** | **Overall**  **(n = 125)** | **Not in a cluster**  **(n = 56)** | **In a cluster**  **(n = 69)** | **Odds ratio** | **95% CI** | ***p*** | **Adjusted odds ratio** | **95% CI** | ***p*** |
| --- | --- | --- | --- | --- | --- | --- | --- | --- | --- |
|  |  |  |  |  |  |  |  |  |  |
| ^*^Shared needle | 18 (17.7) | 5 (11.1) | 13 (22.8) | 2.36 | 0.77-7.22 | 0.131 | 4.85 | 1.15-20.44 | **0.032** |
| ^*^New needle | 78 (73.6) | 36 (78.2) | 42 (70.0) | 0.65 | 0.27-1.58 | 0.341 |  |  |  |
| Age ≥ 29 | 77 (61.6) | 28 (50.0) | 49 (71.0) | 2.45 | 1.17-5.13 | **0.017** | 3.59 | 1.28-10.11 | **0.016** |
| ***Race** |  |  |  |  |  |  |  |  |  |
| White |  |  |  | 1.00 |  |  | 1.00 |  |  |
| Black | 42 (34.7) | 29 (55.8) | 13 (18.8) | 0.28 | 0.12-0.66 | **0.004** |  |  |  |
| Mixed ancestry | 27 (22.3) | 3 (5.8) | 24 (34.8) | 5.00 | 1.33-18.79 | **0.017** | 8.51 | 1.61-44.93 | **0.012** |
| City |  |  |  |  |  |  |  |  |  |
| Durban |  |  |  | 1.00 |  |  | 1.00 |  |  |
| Pretoria | 56 (44.8) | 31 (55.7) | 25 (44.6) | 1.48 | 0.61-3.56 | 0.383 |  |  |  |
| Cape Town | 35 (28.0) | 3 (5.4) | 32 (46.4) | 19.56 | 4.94-77.47 | **0.000** |  |  |  |

* Indicates variables with missing information

19 missing for New needle variable Overall (n = 106), total not in cluster (n = 46), total in cluster (n = 60).

23 missing for Shared needle variable Overall (n = 102), total not in cluster (n = 45), total in cluster (n = 57).

4 missing for Race variable Overall (n= 121), total not in cluster (n = 52), total in cluster (n = 69).

In multivariate analysis, three factors were positively associated with HCV phylogenetic clustering, including sharing a needle at the last injection (aOR 4.85, 95% CI 1.15 - 20.44, p = 0.032), age ≥29 years (aOR 3.59, 95% CI 1.28 - 10.11, p = 0.016), and mixed ancestry race (aOR 8.51, 95% CI 1.61 - 44.93, p = 0.012, **S4 Table**). Sharing a needle at the last injection was associated with clustering when adjusted for age ≥29 years and mixed ancestry race. The odds of being in a cluster were 5 times greater if the participant shared an injecting needle, 4 times greater if the age was ≥29 years, and 9 times greater if the participant was of mixed ancestry. Residing in Cape Town was positively associated with clustering in the univariate analysis and not with the adjusted analysis. Further, sharing a needle at the last injection was not associated with clustering in the univariate analysis but was associated with clustering when adjusted for age ≥29 years and mixed ancestry race. The median age ≥29 years used in this analysis was similar to that of the larger study (11).
