# Supplementary material for: Factors associated with phylogenetic clustering of hepatitis C virus, mainly among people who inject drugs who access HIV prevention services in South Africa, 2016–2017
Source: PLoS One. 2025 Dec 1;20(12):e0336614. doi: 10.1371/journal.pone.0336614 (PMC12668479; doi:10.1371/journal.pone.0336614)
Supplement: Supplementary file 5 [file pone.0336614.s008.docx]

UNIVERSITY OF THE WITWATERSRAND, JOHANNESBURG

R14/49 Mr N Ndlovu

HUMAN RESEARCH ETHICS COMMITTEE (MEDICAL)
CLEARANCE CERTIFICATE NO. M181169

**NAME:** **(Principal Investigator)**	Mr N Ndlovu
**DEPARTMENT:**	School of Pathology<br>Department of Virology<br>National Institute of Communicable Diseases<br>National Health Laboratory Service<br>Sandringham
**PROJECT TITLE:**	Phylogenetic clustering and molecular characterization of hepatitis C in persons who inject/use drugs (PWID/UD's) and men who have sex with men (MSM) in four cities of South Africa
**DATE CONSIDERED:**	30/11/2018
**DECISION:**	Approved unconditionally
**CONDITIONS:**	Sub-Study under M160510
**SUPERVISOR:**	Dr N Prabdial-Sing
**APPROVED BY:**	Dr CB Penny, Chairperson, HREC (Medical)
**DATE OF APPROVAL:**	14/01/2019

This clearance certificate is valid for 5 years from date of approval. Extension may be applied for.

DECLARATION OF INVESTIGATORS

To be completed in duplicate and **ONE COPY** returned to the Research Office Secretary on 3rd floor, Phillip V Tobias Building, Parktown, University of the Witwatersrand, Johannesburg.
I/We fully understand the conditions under which I am/we are authorised to carry out the above-mentioned research and I/we undertake to ensure compliance with these conditions. Should any departure be contemplated from the research protocol as approved, I/we undertake to resubmit to the Committee. **I agree to submit a yearly progress report**. When a funder requires annual re-certification, the application date will be one year after the date of the meeting when the study was initially reviewed. In this case, the study was initially reviewed in **November** and will therefore reports and re-certification will be due early in the month of **November** each year. Unreported changes to the application may invalidate the clearance given by the HREC (Medical).

Principal Investigator Signature

16/01/2019
Date

PLEASE QUOTE THE PROTOCOL NUMBER IN ALL ENQUIRIES
