# Supplementary material for: Factors associated with phylogenetic clustering of hepatitis C virus, mainly among people who inject drugs who access HIV prevention services in South Africa, 2016–2017
Source: PLoS One. 2025 Dec 1;20(12):e0336614. doi: 10.1371/journal.pone.0336614 (PMC12668479; doi:10.1371/journal.pone.0336614)
Supplement: Attachment — Submitted filename: PONE-D-25-15243.docx [file pone.0336614.s007.docx]

**PONE-D-25-15243**

**Research Article** **Title:** Factors Associated with Phylogenetic Clustering of Hepatitis C Virus, Primarily Among People Who Inject Drugs Accessing HIV Prevention Services in South Africa, 2016-2017.

**Author:** Nkosenhle Lindokuhle Ndlovu, M.D.

Thank you for the opportunity to review this research article, which addresses a significant public health problem in South Africa. This study received approval from the University of the Witwatersrand Human Research Ethics Committee (clearance certificate M181169), a copy of which was included in the documents shared for review.

The sample and study data were drawn from a previous study that examined viral infections in people who inject drugs (PWID) and men who have sex with men (MSM) in South Africa (with references to publications from this project shared).

Hepatitis C virus (HCV) predominates in key population groups, with genotypes 1a and 3a being the most common identified in this study. This study effectively uses molecular epidemiology to genetically link related cases across various South African cities.

The study is well written, with clear objectives, study methodology and study findings/discussion.

**Comments, Clarifications, or Queries:**

1. Were there participants with mixed infections, for example, with both 1a and 3a genotypes, possibly due to cross-infections?
2. The shared letter of approval indicates that the study was approved for four cities in South Africa, yet the study itself mentions only three. Please clarify this discrepancy.
3. Regarding Table 1A, what does "N/A" signify when referring to the MSM population for Pretoria and Durban? Does it indicate missing data or no MSM populations recruited into the study?
4. What was the study design for the original study from which the samples for this research were obtained? Additionally, what was the study design for samples acquired from the "specialist clinic in Cape Town"?
5. This study has several acknowledged limitations. It is also worth noting that establishing the precise age of infection in study participants can be challenging or may not be accurate when self-reported.
