# Supplementary material for: Factors associated with phylogenetic clustering of hepatitis C virus, mainly among people who inject drugs who access HIV prevention services in South Africa, 2016–2017
Source: PLoS One. 2025 Dec 1;20(12):e0336614. doi: 10.1371/journal.pone.0336614 (PMC12668479; doi:10.1371/journal.pone.0336614)
Supplement: Attachment — Submitted filename: Response to Reviewers.docx [file pone.0336614.s009.docx]

Dear Editors and Reviewers,

We sincerely thank you for your thoughtful and constructive comments on our manuscript. Below we address each point raised, detailing the revisions made or clarifications provided to improve the quality and clarity of our work.

**Reviewer 1**

1. Page 14, line 116: it is said that samples were collected from PWID, MSM and PWUD populations. However, in the abstract, only PWID and MSM are mentioned as the source of sequences used for phylogenetic clustering. The PWUD inclusion is not explained in the abstract. PWUD stands for?

- Response: PWUD/ID (people who use drugs including people who inject drugs) were recruited from cities that operated harm reduction services at a PWUD (people who use drugs): PWID (people who inject drugs) ratio of 1:4 due to the increased risk of blood borne infection transmission through injecting practices. For this analysis, the majority of samples would have come from PWID, however the PWUD may have included people with previous experiences of injecting, but this was not explicitly assessed.
- The comment is addressed in Lines 116 -112
- Scheibe, A., Young, K., Versfeld, A., Spearman, C. W., Sonderup, M. W., Prabdial-Sing, N., Puren, A., & Hausler, H. (2020). Hepatitis B, hepatitis C and HIV prevalence and related sexual and substance use risk practices among key populations who access HIV prevention, treatment and related services in South Africa: findings from a seven-city cross-sectional survey (2017). BMC Infectious Diseases, 20(1), 655. <https://doi.org/10.1186/s12879-020-05359-y>

2. In the tree of Fig 2. The authors used Smith Panel sequences as reference sequences for phylogenetic analysis. However, the tree appears noisy and difficult to interpret. It would be helpful if the authors could improve the visualization. One possible approach to improve clarity would be to initially use all reference sequences for classification. Once the sequences of interest are grouped with relevant reference clades, a refined tree could be constructed using only the most relevant subset of reference sequences. This would enhance the readability of the final tree while maintaining the accuracy of the phylogenetic relationships.

- **Response:** Thank you for pointing this out. I have reworked this tree into figures 2a and 2b to improve readability. Additionally, Some nodes have been collapsed on figtree to show the most relevant subset of sequences. Addressed in line 266

**Reviewer 2**

1. Were there participants with mixed infections, for example, with both 1a and 3a genotypes, possibly due to cross-infections?

- **Response:** Yes, there were participants with dual infection, although in very small numbers. The assay we used for sequencing is pan genotypic and hence these were excluded from the analysis.
- Addressed in line 126

2. The shared letter of approval indicates that the study was approved for four cities in South Africa, yet the study itself mentions only three. Please clarify this discrepancy.

- **Response:** Yes, correct. The study was approved for 7 studies, and recruitment was done across all of those cities. PWUD/ID were recruited from DBN, CPT, PTA and MSM from CPT, PTA and JHB
- See:Scheibe, A., Young, K., Versfeld, A., Spearman, C. W., Sonderup, M. W., Prabdial-Sing, N., Puren, A., & Hausler, H. (2020). Hepatitis B, hepatitis C and HIV prevalence and related sexual and substance use risk practices among key populations who access HIV prevention, treatment and related services in South Africa: findings from a seven-city cross-sectional survey (2017). BMC Infectious Diseases, 20(1), 655. https://doi.org/10.1186/s12879-020-05359-y

3. Regarding Table 1A, what does "N/A" signify when referring to the MSM population for Pretoria and Durban? Does it indicate missing data or no MSM populations recruited into the study?

- **Response:** The “N/A” indicates no data. MSM were recruited but these participants were not included in the analysis because no sequence data was available. Only the MSM population from the specialist clinic in Cape Town was included.
- Addressed in line 244.

4. What was the study design for the original study from which the samples for this research were obtained? Additionally, what was the study design for samples acquired from the "specialist clinic in Cape Town"?

- **Response:** cross sectional survey that used convenience sampling recruiting from HIV and harm reduction services for key populations in the selected cities. Addressed in line 115
- **Response:** Specialist clinics: convenience sampling from clients accessing the MSM services for HIV, STI and sexual health services at a specialist MSM clinic.
- Addressed in line 128

5. This study has several acknowledged limitations. It is also worth noting that establishing the precise age of infection in study participants can be challenging or may not be accurate when self-reported.

- **Response:** Agreed, and thank you for pointing this out. Addressed in line 429 and 430.

We trust these revisions adequately address the reviewers’ concerns. We appreciate the reviewers' insights that have helped to improve our manuscript.

Sincerely,

Nkosenhle Lindo Ndlovu
